# Out-of-clinic measurement of sweat chloride using a wearable sensor during low-intensity exercise

**DOI:** 10.1038/s41746-020-0257-z

**Published:** 2020-03-27

**Authors:** Dong-Hoon Choi, Grant B. Kitchen, Mark T. Jennings, Garry R. Cutting, Peter C. Searson

**Affiliations:** 1 Institute for Nanobiotechnology, John Hopkins University, Baltimore, MD USA; 2 Department of Medicine, Division of Pulmonary and Critical Care, Johns Hopkins Hospital, Baltimore, MD USA; 3 Institute of Genetic Medicine, Johns Hopkins University, Baltimore, MD USA; 4 Department of Materials Science and Engineering, Johns Hopkins University, Baltimore, MD USA

**Keywords:** Biomedical engineering, Sensors

## Abstract

Wearable sensors have the potential to enable measurement of sweat chloride outside the clinic. Here we assess the feasibility of mild exercise as an alternative to pilocarpine iontophoresis for sweat generation. The results from this proof-of-concept study suggest that mild exercise could be a feasible approach to obtain reliable measurements of sweat chloride concentration within 20–30 min using a wearable sensor.

## Introduction

Sweat chloride is a biomarker for cystic fibrosis (CF)^1^. Sweat tests involve sweat induction, usually by pilocarpine iontophoresis, and measurement of the sample using an analytical instrument. Recent advances in wearable sensors could enable measurement of sweat chloride outside an approved CF clinic^2,3^, of which there are only 130 in the United States, potentially reducing the need to travel and enabling individuals on CF transmembrane conductance regulator modulator therapy to track their sweat chloride at home for precise clinical management. While wearable sensors could replace laboratory-based analytical measurements, sweat induction outside the clinic is a major challenge since iontophoresis presents challenges for home-based use.

Mild exercise is an alternative method for sweat induction outside the clinic. Even though the exercise capacity of individuals with CF may be impaired, regular exercise is recommended to improve lung function and survival^4,5^. In this work, we tested the hypothesis that walking or slow jogging in moderate relative humidity (47.1 ± 17.0%) and at room temperature (24.9 ± 0.2 °C) could be used to generate sufficient sweat for measurement with a wearable sweat sensor within a reasonable time frame. We addressed three key issues: (1) the onset time for sweating under low-intensity exercise, (2) the time needed to make a measurement of sweat chloride using a wearable sensor, and (3) comparison of sensor measurements to conventional laboratory measurements. First, we assessed the sweat chloride profiles of five healthy individuals while walking or jogging at 1.8, 2.0, and 2.2 m s^−1^ and simultaneously measured the sweat rate. Next, we compared the sweat chloride results from the sensor to standard laboratory analysis following pilocarpine iontophoresis. Finally, we assessed the intra-individual variation in repeated measurements using the wearable sensor. The results suggest that using a wearable sensor during mild exercise is a feasible approach for measurement of sweat chloride.

## Results and discussion

### Onset time for sweating, sweat rate, and sweat concentration

**Fig. 1:**
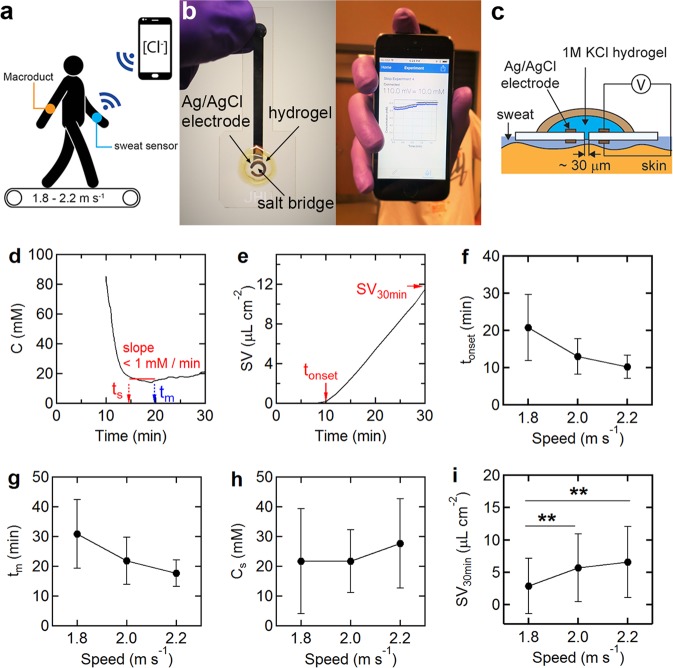
Sweat test using a wearable sweat chloride sensor during walking. **a** Measurement of sweat chloride concentration and sweat volume using a wearable sweat chloride sensor and Macroduct device. **b** Optical images of the sweat sensor and the smartphone app. **c** Schematic illustration of the wearable potentiometric sensor. **d** Representative sweat chloride concentration profile for an individual walking at 1.8 m s^−1^. **e** Corresponding sweat volume versus time curve. **f** The time corresponding to the onset of sweating (*t*_onset_) (mean ± SD). **g** The minimum measurement time (*t*_m_). **h** The sweat chloride concentration measured by the sensor. *C*_s_ is the average value obtained from *t*_s_ to the end of the trial. **i** Sweat volume per unit area collected in a Macroduct over 30 min from the beginning of the trial. **P < 0.01.

To assess the feasibility of mild exercise for sweat induction outside the clinic, five subjects were asked to walk or jog on a treadmill at 1.8, 2.0, and 2.2 m s^−1^ (Fig. 1a). This is slightly faster than the range (95% CL) of average walking speeds for healthy adults of about 1.1–1.5 m s^−1^ ^6^. All five individuals walked at 1.8 m s^−1^ and jogged at 2.2 m s^−1^. The sweat chloride concentration was measured using a wearable sensor and the sweat rate was measured using a Macroduct sweat collection device (Fig. 1b, c)^3^. Within a few minutes after the onset of sweating, the sensor output stabilized (Fig. 1d). The onset of sweating (*t*_onset_) was defined as the time at which sweat was visually observed in the Macroduct coil (Fig. 1e). The sensor stabilization time (*t*_s_) and the minimum measurement time (*t*_m_) were defined by the start and end times at which a 5 min moving average window had a slope (linear least-squares fit) of <1 mM min^−1^ (Fig. 1d).

At 1.8 m s^−1^, all subjects started sweating within 20.8 ± 8.9 (SD) min (Fig. 1f) and the measurement time was 30.8 ± 11.5 min from the beginning of the trial (Fig. 1g). At 2.2 m s^−1^, the measurement time decreased to 17.6 ± 4.4 min. The sweat chloride concentrations for the five individuals were independent of ambulation speed, indicating that reproducible measurements can be achieved at any fast walking or slow jogging speed (Fig. 1h). At higher exercise intensities the sweat rate may become sufficiently large that the sweat chloride concentration increases above this baseline^7^. The cumulative sweat volume after 30 min (SV_30 min_) at 1.8, 2.0, and 2.2 m s^−1^ were 2.9 ± 4.3, 5.7 ± 5.2, and 6.6 ± 5.5 μL cm^−2^, respectively (Fig. 1i). We estimate the minimum sweat volume for detection to be about 0.6 μL cm^−2^ (see Supplementary Fig. S1). The average sweat volume at *t*_s_ for all trials was 1.0 ± 1.2 μL cm^−2^. Experimentally, the sweat volume at *t*_s_ could be defined as a criteria for sensor detection.

### Comparison walking-induced sweating to chemically induced sweating

To compare the walking tests to standard laboratory tests, we performed trials on 11 healthy subjects walking at 1.8 m s^−1^ and compared the sweat chloride concentration from the sensor (*C*_s_) to the sweat chloride concentration obtained following pilocarpine iontophoresis (*C*_pi_) (Table 1). The average values of *C*_s_ and *C*_pi_ were 16.9 ± 12.5 and 22 ± 11.9 mM, respectively, and there was no statistical difference. The standard deviations for both methods are the same but slightly larger than the values of 5–7 mM reported for variation in pilocarpine measurements between forearms on the same individual^8,9^.

**Table 1:** Comparison of sweat chloride concentration obtained following pilocarpine iontophoresis or during walking at 1.8 m s^−1^.

Subject	*C*_pi_ (mM)	*C*_s_ (mM)	*C*_ex_ (mM)	*t*_m_ (min)
1	10	11.8	19	50.9
2	10	8.2	<10	20.5
3	<10	4.7	<0	18.1
4	34	49	52	26.6
5	35	16.9	QNS	45.5
6	35	21.1	QNS	36.9
7	11	8.4	<0	20.4
8	17	15.8	16	34.1
9	24	36.8	19	21.6
10	16	5.8	<10	27.6
11	14	18.6	13	26.7

*C*_*pi*_ standard sweat test using pilocarpine iontophoresis, *C*_*s*_ sweat sensor during walking at 1.8 m s^−1^, *C*_ex_ sweat sample collected by a Macroduct during walking at 1.8 m s^−1^, *QNS* quantity not sufficient (sweat volume <15 μL).

We next compared sensor results (*C*_s_) to standard laboratory tests of samples collected during exercise using a Macroduct collection device (*C*_ex_). Apart from two QNS results and subject 9, *C*_ex_ values were in good agreement with sensor results (*C*_s_). To assess sensor accuracy, we compared sensor calibration curves (sensor voltage vs. chloride concentration) recorded before and after all trials (see Supplementary Table S1). The maximum sensor error caused by changes in calibration curves before and after trials was 1.95 ± 2.99 (SD) mM, and hence sensor error does not contribute significantly to the variation between *C*_s_ and *C*_pi_.

Next, the sweat chloride concentrations obtained from the sensor were compared to laboratory analysis of samples collected during the walking trial. For 6 of 11 subjects, either the volume of sweat collected was too small for laboratory measurement (≤15 µL), or the concentration was below the threshold for measurement (≤10 mM). The concentration threshold would likely not be an issue for individuals with CF. The values of *C*_ex_ for the five subjects with recorded values were in good agreement with sensor readings. The average measurement time for 11 subjects walking at 1.8 m s^−1^ was 29.7 ± 10.9 min.

### Variation in walking-induced sweating

To assess the daily variation in the sweat chloride concentration obtained by the wearable sensor during walking, three healthy subjects repeated walking trials at 1.8 m s^−1^ on 5 days. Before each walking trial, the sweat chloride concentration was also measured using a standard sweat test. Subjects 1 and 2 had coefficients of variation (CV) of 7% and 11% for the walking trials, respectively (Table 2). The CV for the standard test was 20% for subject 1, but could not be measured for subject 2 since some values were below the concentration threshold. Subject 3 had a CV of 44% during the walking trials, compared to a value of 23% for the standard sweat test. The values of CV for within-subject variation in sweat chloride from both sensor measurements and pilocarpine sweat tests are consistent with values of 10–45% previously reported for standard pilocarpine sweat tests (8 repeated trials for 12 individuals)^10^.

**Table 2:** Variation of sweat chloride concentration measured by a wearable sensor during walking at 1.8 m s^−1^ and from a standard sweat test following pilocarpine iontophoresis.

Subject	Trials	Sweat sensor test			Standard sweat test		
		Median (mM)	Range (mM)	CV (%)	Median (mM)	Range (mM)	CV (%)
1	5	17	15.6–18.6	7	16	13–21	20
2	5	5.7	4.9–5.8	11	10	<10–16	N/A^a^
3	5	25.1	8.1–36.8	44	24	21–31	23%

^a^The sweat concentration for two of five results was less than the threshold for laboratory measurement (10 mM).

In conclusion, the results from this proof-of-concept study suggest that mild exercise could be a feasible approach to obtain reliable measurements of sweat chloride concentration within 20–30 min using a wearable sensor.

## Methods

### On-body trials

All on-body trials were performed under a protocol approved by the Institutional Review Board (IRB) at Johns Hopkins University (IRB00134667). All participants provided written informed consent before participation. Participants read the study participation informative document and signed the corresponding informed consent. To assess the influence of speed on sweat chloride and sweat rate, five healthy subjects (four males, one female) were asked to walk or jog on a treadmill (Cybex 530 T) at 1.8, 2.0, and 2.2 m s^−1^ (Fig. 1a). The subjects consumed no food or water in the 3 h before the trial and 5 mL kg^−1^ of bodyweight water was provided before the start of each trial. To measure the sweat chloride concentration and sweat volume, we used a wearable sweat sensor (Fig. 1b, c) and a Macroduct (Wescor) sweat collection device^2,3^. Details of sensor design and performance are provided in Supplementary Fig. S2. To determine sensor stabilization time (*t*_s_) and the sweat volume collected in the Macroduct device, customized MATLAB and LabVIEW codes were employed, respectively^3,11^. The sensors were attached to the forearm of each subject using a commercial adhesive bandage (Nexcare, Tegaderm). All trials were performed at constant temperature (24.9 ± 0.2 °C) and relative humidity (47.1 ± 17.0%), and identical t-shirt and shorts were provided to all participants.

To compare the walking tests to standard sweat tests, 11 healthy subjects first completed a conventional laboratory sweat test. Sweat was induced by pilocarpine iontophoresis on the left forearm (Wescor, Model 3700) and collected using a Macroduct for 30 min^1^. The collected sweat sample was transferred to an air-tight tube and stored in a refrigerator. Within 1 h after the conventional sweat test, subjects completed a walking trial on the treadmill at 1.8 m s^−1^ with a wearable sensor on the right forearm. Subjects were asked to walk for 30 min but walked for longer if the sweat volume collected in Macroduct was <15 μL. The trials were terminated regardless of the collected sweat volume whenever the subjects wanted to stop walking. During the walking trials, a sweat sample was collected using a Macroduct collection device on the left forearm, but at a different location from where iontophoresis was performed. Sweat samples (obtained from pilocarpine iontophoresis and exercise) were analyzed at the Johns Hopkins Chemical Core Laboratory.

### Reporting summary

Further information on research design is available in the Nature Research Reporting Summary linked to this article.

## Supplementary information

Supplementary Information

Reporting Summary

## Data Availability

The data that support the findings of this study are available from the corresponding author upon request.

## Code availability

The customized MATLAB (MATLAB 2018) and LabVIEW (LabVIEW 2016 with the Vision Development Module or LabVIEW Run-Time Engine 2016) codes to analyze sensor stabilization time and the sweat volume collected in the Macroduct devices are available from the corresponding author upon request.
