# Supplementary material for: Out-of-clinic measurement of sweat chloride using a wearable sensor during low-intensity exercise
Source: NPJ Digit Med. 2020 Mar 27;3:49. doi: 10.1038/s41746-020-0257-z (PMC7101332; doi:10.1038/s41746-020-0257-z)
Supplement: Supplementary file 1 — Supplementary Information [file 41746_2020_257_MOESM1_ESM.pdf]

Supplementary Information

Out-of-clinic measurement of sweat chloride using a wearable sensor during low intensity exercise

Dong-Hoon Choi,[a] Grant Kitchen,[a] Mark T. Jennings,[b] Garry R. Cutting[c] and Peter C. Searson[*a,d]

[a] Institute for Nanobiotechnology, John Hopkins University, Baltimore, MD, USA

[b] Division of Pulmonary and Critical Care, Department of Medicine, Johns Hopkins Hospital, Baltimore, MD, USA

[c] Institute of Genetic Medicine, Johns Hopkins University, Baltimore, MD, USA

[d] Department of Materials Science and Engineering, Johns Hopkins University, Baltimore, USA

Figure S1. Estimation of the minimum sweat volume for sensor detection

Table S1. Sensor error due to drift in calibration curves

Figure S2. Wearable sweat sensor

Estimation of the minimum sweat volume for sensor detection

We estimate the minimum volume for sensor detection of the sweat chloride concentration (≈ 0.64 μL) in the following way.

(1) We assume that skin has 40 μm deep parallel grooves. If each groove is taken to be an equilateral triangle, then the cross-sectional area is 1600 μm^2.
(2) We assume a line density of grooves of 40 cm^{-1}, so that the volume in the grooves per cm^2 is about 0.64 μL

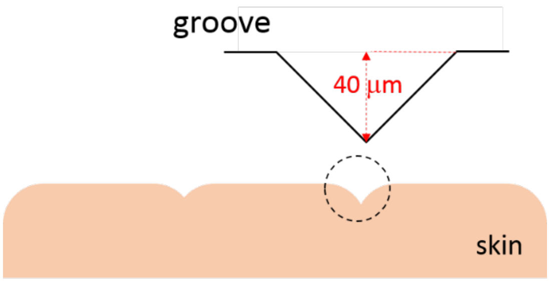

Supplementary Figure 1. Schematic illustration of the grooves in skin which are typically 40 μm in depth.

Sensor error due to drift in calibration curves

Sensor calibration was performed in the following way: (1) the working electrode was rinsed in deionized (DI) water for 40 s, (2) 100 μL of 10 mM NaCl (Fisher Scientific) solution was placed on the working electrode of the sensor using a micropipette, (3) the sensor voltage was measured and recorded for 3 minutes, (4) steps 1 - 3 were repeated with 50 and 100 mM NaCl solutions, (5) the sensor voltage at each concentration was determined by averaging the recorded voltages over last 1 min, and (6) using a linear least squares fit to V vs. log C), the relationship between the measured voltage and the concentration of the test solution was established. All calibrations were performed at room temperature. For on-body use, the sensor calibration curves were adjusted to 32 ˚C using the Nernst equation.

To evaluate the accuracy during walking trials, all sensors were calibrated before and after each trial and the sensor error (ΔC_S) due to changes in the calibration curves was calculated from:

$$\Delta C_s = \frac{1}{n \cdot (t_{end} - t_s)} \cdot \left(\sum_{t=t_s}^{t_{end}} 10^{\frac{V(t)-y_a}{m_a}} - \sum_{t=t_s}^{t_{end}} 10^{\frac{V(t)-y_b}{m_b}} \right)$$

where t_s is the sensor stabilization time, t_{end} is the end time of the trial, n is the sampling rate of the wireless transceiver per minute, $V(t)$ is the output voltages of the sensor during the trials, m is the slope of the calibration curve (mV/decade), and y is the y-intercept (mV) of the calibration curve of each sensor. The subscripts of b and a denote the calibrations before and after the trials, respectively. The units of t_s and t_{end} are minutes, and $n = 60\ \text{min}^{-1}$.

Supplementary Table S1. Slope m and y-intercept y of calibration curves before and after trials. C_s (mM) is the measured value of sweat chloride based on the calibration curve from before or after the trial. ΔC (mM) is the difference in sweat chloride ion concentration based on the calibration curves obtained before and after each trial. m (mV/decade), y (mV).

subject	before			after			ΔC
	m_b	y_b	C_s	m_a	y_a	C_s	(after – before)
**1**	-59.0	204.4	11.8	-57.1	201.9	11.6	-0.2
**2**	-55.9	184.9	8.2	-54.7	183.8	8.2	0
**3**	-57.8	173.5	4.7	59.2	180.1	5.8	1.1
**4**	-57.6	200.9	49.0	-57.0	203.7	57.2	8.2
**5**	-54.1	171.5	36.8	-60.9	183.9	39.3	2.5
**6**	-58.1	201.7	17.4	-59.7	204.6	17.9	0.5
**7**	-57.0	173.3	21.1	-55.9	175.6	24.9	3.8
**8**	-54.9	167.6	5.8	-54.8	177.9	8.9	3.1
**9**	-52.6	185.8	18.6	-57.9	198.5	23.6	5.0
**10**	-54.9	167.6	8.4	-54.8	177.9	8.7	0.3
**11**	-62.9	212.8	15.8	-59.3	203.2	13.0	-2.8

Wearable sweat sensor

Sweat profiles were recorded using a potentiometric wearable chloride ion sensor developed in our lab [1-4]. The sensor was fabricated on a flexible PET (Polyethylene terephthalate) substrate using a conventional physical deposition process and a laser drilling process. The salt bridge was designed to minimize equilibration between the sweat sample and the reference solution. The sensor reliably measures sweat chloride concentration over 12 hours with error less than 4 mM, and the response time is about 2 s.

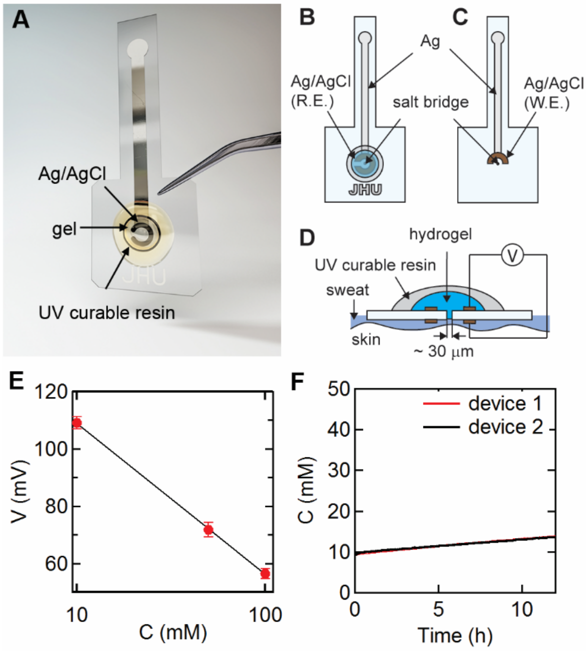

Supplementary Figure S2. Wearable sweat chloride sensor. (A) Optical image of a sensor after fabrication. Schematic illustration of the sensor: (B) top side (reference electrode), (C) bottom side (working electrode), and (D) cross-section. (E) Sensor calibration curves (N = 6). Data represent mean ± SD. (F) Measured chloride ion concentration over 12 hours in 110 μL of 10 mM NaCl. The drift rate due to equilibration was 0.3 mM h^{-1} over 12 hours.

References

1. D. H. Choi, A. Thaxton, I. C. Jeong, K. Kim, P. R. Sosnay, G. R. Cutting and P. C. Searson. Sweat test for cystic fibrosis: Wearable sweat sensor vs. standard laboratory test. *J Cyst Fibros* 17, e35-e38 (2018).
2. D. H. Choi, Y. Li, G. R. Cutting and P. C. Searson. A wearable potentiometric sensor with integrated salt bridge for sweat chloride measurement. *Sensors and Actuators B-Chemical* 250, 673-678 (2017).
3. D. H. Choi, G. Kitchen, J. S. Kim, Y. Li, K. Kim, I. C. Jeong, J. Nguyen, K. J. Stewart, S. L. Zeger and P. C. Searson. Two Distinct Types of Sweat Profile in Healthy Subjects While Exercising at Constant Power Output Measured by a Wearable Sweat Sensor. *Sci Rep* 9, 17877 (2019).
4. D. H. Choi, J. S. Kim, G. R. Cutting and P. C. Searson. Wearable Potentiometric Chloride Sweat Sensor: The Critical Role of the Salt Bridge. *Anal Chem* 88, 12241-12247 (2016).
